# ConTEdb: a comprehensive database of transposable elements in conifers

**DOI:** 10.1093/database/bay131

**Published:** 2018-12-17

**Authors:** Fei Yi, Juanjuan Ling, Yao Xiao, Hanguo Zhang, Fangqun Ouyang, Junhui Wang

**Affiliations:** 1 State Key Laboratory of Tree Genetics and Breeding, Key Laboratory of Tree Breeding and Cultivation of State Forestry Administration, Research Institute of Forestry, Chinese Academy of Forestry, Beijing, China; 2 College of Biological and Pharmaceutical Sciences, Three Gorges University, Yichang, China; 3 State Key Laboratory of Tree Genetics and Breeding, Northeast Forestry University, Harbin, China

## Abstract

Conifers are the largest and most ubiquitous group of gymnosperms and have significant ecological significance and economic importance. However, the huge and complex genomes have hindered the sequencing and mining of conifer genomes. In this study, we identified 413 423 transposable elements (TEs) from *Picea abies*, *Picea glauca* and *Pinus taeda* using a combination of multiple approaches and classified them into 11 133 families. A comprehensive web-based database, ConTEdb, was constructed and served for researchers. ConTEdb enables users to browse, retrieve and download the TE sequences from the database. Several analysis tools are integrated into ConTEdb to help users mine the TE data easily and effectively. In summary, ConTEdb provides a platform to study TE biology and functional genomics in conifers.

## Introduction

Transposable elements (TEs) are DNA sequences that have the ability to integrate into the genome at a new site within their cell of origin (1). They contribute greatly to eukaryotic genomes, particularly plant genomes, owing to their ability to increase copy number in the process of transposition (2). TEs are classified into two classes, retrotransposon and DNA transposon, based on their transposition mechanisms (3). Retrotransposons are transcribed into RNA and then reverse transcribed and reintegrated into the genome, which is the so-called ‘copy and paste’ mechanism. Unlike retrotransposon, DNA transposons are generally excised from one genomic site and integrated into another by the ‘cut and paste’ mechanism. Within each class, TEs are further subdivided into orders, superfamilies and families on the basis of the structural and enzymatic criteria (3).

Although they are often considered as ‘junk DNA’, more and more evidence demonstrates that TEs not only contribute to the shaping of genomes through their amplification and recombination (4) but also play significant roles in regulating the expression of genes (5, 6) and creating the raw material for the evolution of new genes and new genetic functions (7, 8).

Conifers (Coniferales) are the largest and most ubiquitous group of gymnosperms and are placed in 6 families, 69 genera and 605 species (9). They are woody perennials that shape many northern hemisphere ecosystems and support large industries through the provision of wood, fiber and energy. Sequencing conifer genomes is relevant because of their taxonomic position, ecological significance and economic importance. However, conifer genomes are extremely large and contain considerable amounts of repetitive DNA, especially transposons, which is a huge challenge for genome sequencing and assembly (10, 11). Therefore, the precise identification and classification of TEs at the whole genome level are very important. Three conifer genomes, *Picea abies* (12), *Picea glauca* (13, 14) and *Pinus taeda* (15–17), have been sequenced so far. Researchers can obtain the TE information of them and other conifers from some databases, such as CGN (Conifer Genomics Network), ConGenIE and Repbase, but there are limitation in number. In this study, TEs in the genomes of the sequenced conifers were identified and classified by a combined approach. All identified TEs were deposited in the conifer TE database, ConTEdb, and some tools were integrated into it to facilitate the usage. As thus, ConTEdb provides a platform to study TE biology and functional genomics in conifers.

**Table 1:** List of conifers analyzed in this study

**Plant species**	**URL**
*P. abies*	ftp://ftp.ncbi.nlm.nih.gov/genomes/all/GCA/900/067/695/ (GCA_900067695.1_Pabies01/GCA_900067695.1_Pabies01_genomic.fna.gz)
*P. glauca*	ftp://ftp.ncbi.nlm.nih.gov/genomes/all/GCA/000/411/955/ (GCA_000411955.5_PG29_v4.1/GCA_000411955.5_PG29_v4.1_genomic.fna.gz)
*P. taeda*	ftp://ftp.ncbi.nlm.nih.gov/genomes/all/GCA/000/404/065/ (GCA_000404065.3_Ptaeda2.0/GCA_000404065.3_Ptaeda2.0_genomic.fna.gz)

## Database construction and content

### Data sources

The ConTEdb houses the information on TEs from three conifers, including *P. abies*, *P. glauca* and *P. taeda*. The download address for the assembly genome sequences of the three conifers are listed in Table 1.

### Identification of TEs in the three conifers

A combination of multiple approaches was employed to identify TEs in the three conifers. (i) Signature-based identification of TEs. LTR_FINDER (v 1.05) (18) and MGEScan-nonLTR (v 2.0) (19) programs were used with default parameters to search against the three conifer genomes to identify the LTR (long terminal repeat) and non-LTR retrotransposons, respectively. For Helitron and MITE transposons, HelitronScanner (v 1.1) (20) and MITEHunter (v 20100819) (21) with default parameters were employed to search three assemblies. (ii) Similarity-based identification of TEs. Using RepeatMasker (v 4.0, default parameters; http://www.repeatmasker.org), the genomes of the three conifers were searched against Repbase database for further similarity-based identification of TEs. The results were filtered in line with the criterion that scores <250 or target coverage <40% were removed. (iii) *De novo* identification of TEs. The genomes of the three conifers were analyzed by RepeatScout (v 1.0.5) (22), PILER (v 1.0) (23) and RepeatModeler (v 1.0.7; http://www.repeatmasker.org/RepeatModeler.html), then the putative transposons that have >90% sequence similarity to each other were discarded. For reducing the redundancy, the putative TEs with >90% sequence similarity to the predictions obtained from above two methods were removed.

### Definition of superfamily and families of putative TEs

For each conifer, the putative TEs generated by the above approaches were integrated into a library for definition. In this study, we adopted the criteria of definition proposed by Wicker *et al* (3).

The putative TEs were compared with Repbase database using RepeatMasker (v 4.0, default parameters). The best hit target sequence was selected as the superfamily of the analyzed TEs. Based on the 80-80-80 rule (3) (two elements belonged to the same family if they shared at least 80% of the sequence identity in at least 80% of their coding or internal domain, or within their terminal repeat region, or in both. Meanwhile, in order to prevent misclassification of short and possibly random stretches of homologous sequences, the shortest sequence should be longer than 80 bp.), the TEs of each superfamily were subdivided into different families.

In order to exclude the false positive, the TEs sequences of those superfamilies with <3 families in ConTEdb were extracted as query sequences, and Blastn (1e-5) was performed on the query and Repbase database (subject). In the optimal alignment, the query sequences with coverage <80% were discarded.

**Table 2:** Summary of identified TEs in three conifer genomes

**Class**	**Order**	**Superfamily**	***P. abies* members/families**	***P. glauca* members/families**	***P. taeda* members/families**
Retrotransposons	LTR	*Caulimovirus*	51/17	196/43	315/35
		*Copia*	7304/78	35 826/89	40 645/92
		*Copia(Xen1)*			26/5
		*DIRS*	76/51	226/113	287/143
		*ERV*			7/3
		*ERV1*	299/124	749/231	1392/182
		*ERV4*	25/21	44/39	145/46
		*ERVK*	153/88	563/187	584/169
		*ERVL*	28/22	105/71	115/75
		*Gypsy*	12 267/129	64 831/396	58 349/113
		*Ngaro*	29/24	63/41	126/63
		*Pao*	241/84	832/229	874/179
		RUnknown	9225/941	42 750/3435	43 951/1147
	LINE (long interspersed nuclear element)	*L1*	5230/83	11 150/40	24 553/27
	*PTE-X*	4/4		
	*Tad1*	4/4		
Subtotal			34 936/1670	157 335/4914	171 369/2279
DNA transposons	TIR (terminal inverted repeat)	*hAT*	8/8	12/12	7/7
	*TcMar*	3/3		3/3
		*PIF-Harbinger*		3/3	4/4
		*CMC*			3/3
		DUnknown	7/7	12/12	2/2
	MITE	*MITE*	378/277	287/261	390/297
	Helitron	*Helitron*	6575/609	21 869/359	20 220/403
Subtotal			6971/904	22 183/647	20 629/719
Total			41 907/2574	179 518/5561	191 998/2998

## Results

### Identification of TEs in the three conifers

Using the approaches described earlier, a total of 413 423 TEs belonging to 11 133 families were identified in the three conifer genomes, and the complete result is presented in Table 2. These TEs and families were organized into a web-based database, ConTEdb. In *P. abies* genome, 41 907 transposons were identified, much less than those of *P. glauca* and *P. taeda*, and also the least number of TE families. There were fewer TEs in *P. glauca* than in *P. taeda*, but the number of TE families was nearly twice that of *P. taeda*. Two types of transposons were identified in the three plants, most of which were retrotransposons, and the proportion of DNA transposons was no >20%, which were 16.63 (*P. abies*), 12.36 (*P. glauca*) and 10.74% (*P. taeda*). About 22–24% of TEs were not fully annotated (unknown). Almost all of them were retrotransposons.

For the verification of the identification results, please refer to our previous study (24). The possible false positive rate of the results in ConTEdb was evaluated by randomly extracting 10% of the TEs (including all class/order/superfamily) for copy number analysis. With 10 (copy number) as the threshold, only 0.20 (*P. abies*), 0.77 (*P. glauca*) and 0.53% (*P. taeda*) of predicted TEs were possible false positives.

**Figure 1:**
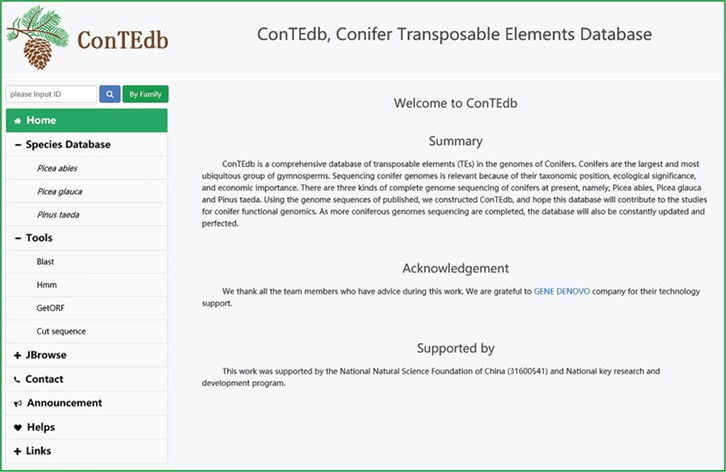
ConTEdb organization and the functional sections in the database.

**Figure 2:**
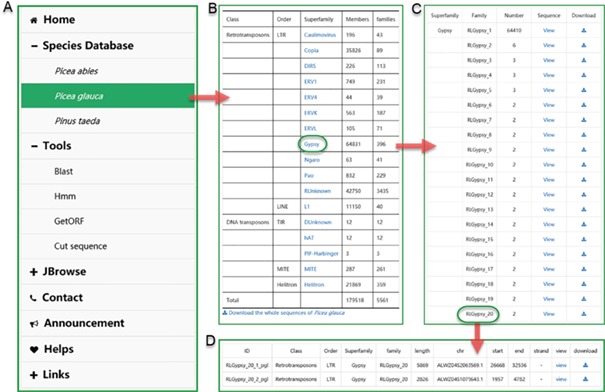
The user interface of browsing in ConTEdb. **(A)** The interface of ‘Species Database’. **(B–D)** Some samples of browsing.

### User interface

ConTEdb is a comprehensive conifer TE database that provide an efficient platform to study TEs in conifers. The web interface was organized into functional sections, and users can browse, search, download and analyze the TE data (Figure 1).

### Browse

In the ‘Species Database’ interface, users can browse the basic information of the TEs in a selected plant species (Figure 2A). By clicking the hyperlink of the species name, the summary of TE information in the form of table is provided to users (Figure 2B). The detailed information of each superfamily can be retrieved by clicking the corresponding entry (Figure 2C). Finally, the exhaustive information of every member of a family, including ID, classification, length, location and nucleotide sequence, are displayed in the corresponding page (Figure 2D).

### Search and download

**Figure 3:**
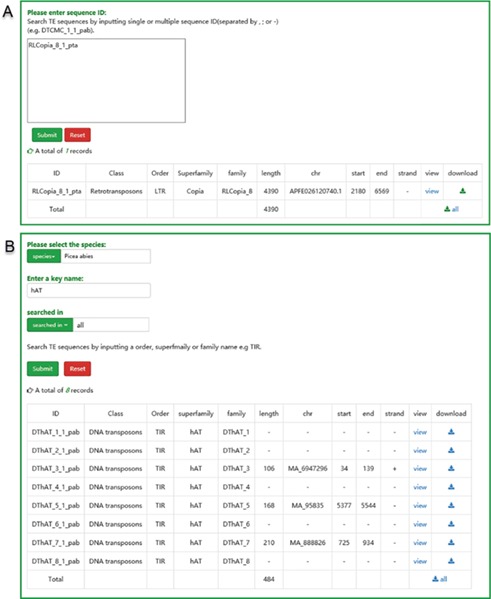
The user interface of searching in ConTEdb. **(A)** The interface of ‘search by ID’ and the result of a sample. **(B)** The interface of ‘search by family’ and the result of a sample.

There are two pathways, namely, ‘search by ID’ and ‘search by family’, available to users for searching. Users can use a specific sequence ID to search the ConTEdb and find the relevant entry (Figure 3A). When employing the second method, users should select one species first and enter a keyword afterward (order, superfamily or family name of TEs), then all the TEs that contain the keyword will be displayed in a tabular format (Figure 3B). The search results can be downloaded by clicking the hyperlinks provided on the page (Figure 3). Furthermore, the TE sequences can also be downloaded in browse page (Figure 2B–D).

### Tools

ConTEdb offers four sequence analysis tools, Blast, Hmm, GetORF and Cut sequence, to facilitate users to analyze the TE data (Figure 1). Using Blast, users can handy and quick comparison of their sequences with the TEs deposited in ConTEdb. The potential open reading frame of the query sequences can be found by GetORF and then search protein sequences against TE profile HMM database. HMMER is provided to facilitate the identification and classification of TEs. As for Cut sequence, it is a tool to extract the sequence in the location defined by users.

### Links

A variety of links to other database and software websites relevant to ConTEdb were included in the main interface (Figure 1).

## Discussion

Conifers possess relatively large genomes compared to most of other land plant species. According to the Gymnosperm DNA C-values database (http://www.kew.org/cvalues/), the genomes of 141 pine plants are as high as 9.5–36 Gb, with an average of 23.68 Gb, which is 190 times that of *Arabidopsis thaliana* and 49 times that of *Populus trichocarpa* (25). Unlike in angiosperms, conifers are not thought to have undergone recent genome duplication event [do not exclude the possibility of paleopolyploidy; (10, 12, 26)]. The huge genome size of conifer seems to result from the slow and steady accumulation of a diverse set of LTR retrotransposons (12).

Studies have indicated that most of the sequences in conifer genomes are repetitive sequences. For example, >99% of the components in *P. abies* genome are moderately or highly repetitive sequences (27) and 70% of them are high-copy repeat contents (12). Transposable elements are the main types of repetitive sequences in conifer, and the number of DNA transposons is limited compared to the retrotransposons (10, 12, 28–30). More than 80% of the TEs in ConTEdb are retrotransposons, especially for *P. taeda* with a proportion of 89.26%. The number of DNA transposons in conifers is limited compared to the retrotransposons, probably due to the lack of effective retrotransposon elimination mechanisms in conifers (12). In ConTEdb, LTR retrotransposons comprised the most abundant fraction of the TEs (70.87–81.43%), with the *Gypsy* superfamily being more abundant than the *Copia* superfamily. Such as there are 35 826 *Copia* retrotransposons in *P. glauca*, while the number of *Gypsy* retrotransposons is 64 831, with a ratio of 1:1.81. This is similar to the results of Nystedt (12) and Morse (31). Compared to *P. glauca* and *P. taeda*, the TEs identified in *P. abies* are much fewer, which may be related to the poor quality of its genome assembly. The scaffold N50 for genome assembly of *P. glauca* and *P. taeda* are 54 661 (NCBI: assembly PG29_v4.1) and 107 038 (NCBI: assembly Ptaeda2.0), respectively, while it is only 2976 for *P. abies* (NCBI: assembly Pabies01), <10% of the previous two. In contrast to angiosperms, the repetitive sequences in conifer genomes are highly diverged and ancient (31–33). The results of Nystedt (12) showed that, in a manually curated library of repetitive sequences, >86% of LTR retrotransposons remained as singletons, indicating that they are quite divergent and that there are several low-abundance families. In our database, there are also lots of low-abundance families. Most of these families even have only one member, especially in ‘unknown’ transposons. For example, in the ‘RUnknown’ superfamily of *P. glauca*, only 311 families contain multiple TEs, and the remaining 3124 are single-member families. This may be the reason of the discrepancy between the number of TEs and the number of families among the three conifers.

There are many databases that contain conifer TEs at present, such as Repbase, PGSB-REcat, ConGenIE and CGN. However, TE data of conifers in these databases are insufficient. For example, there are only 22 TEs of Coniferales in PGSB-REcat, and for Repbase, only 272 related TEs (244, *P. abies*; 2, *P. glauca*; 26, *P. taeda*) are collected in it. As for ConGenIE or CGN and so on, they are not professional transposon databases but have mainly focused on genome data. We established ConTEdb under the infrastructure of the published conifer genome sequences. Compared with existing databases, ConTEdb provides detailed information for TEs in the three conifers, and other databases can use these data to develop their specific functions. Because of the complexity and severe divergent of TEs in conifers, 9232 (*P. abies*), 42 762 (*P. glauca*) and 43 953 (*P. taeda*) transposons (Table 2) were not accurately classified in ConTEdb. We will strive to solve this issue by improving methods and drawing on the research results of others.

## Conclusion

ConTEdb is a database currently consisting of 413 423 TEs in the three conifer genomes along with the classification information. This database provides researchers with not only TE information but also tools for data analysis. With the completion of more conifer genomes sequencing and the improvement of the existing genome assemblies, we commit to continuously update and improve ConTEdb, and the submissions of new data from other researchers are encouraged. Therefore, ConTEdb will be a valuable platform for research into TEs in conifer genomes.

## Notes

**Database URL**: http://genedenovoweb.ticp.net:81/conTEdb/index.php
